# Proteolysis and inflammation of the kidney glomerulus

**DOI:** 10.1007/s00441-021-03433-8

**Published:** 2021-04-17

**Authors:** Fatih Demir, Anne Troldborg, Steffen Thiel, Moritz Lassé, Pitter F. Huesgen, Nicola M. Tomas, Thorsten Wiech, Markus M. Rinschen

**Affiliations:** 1 Department of Biomedicine, Aarhus University, Aarhus, Denmark; 2 Department of Rheumatology, Aarhus University Hospital, Aarhus, Denmark; 3 III. Department of Medicine, University Medical Center Hamburg-Eppendorf, Hamburg, Germany; 4 Christchurch Heart Institute, University of Otago, Christchurch, New Zealand; 5 Forschungszentrum Jülich, Jülich, Germany; 6 CECAD, Medical Faculty and University Hospital, University of Cologne, Cologne, Germany; 7 Nephropathology Section, Institute of Pathology, University Hospital Hamburg Eppendorf, Hamburg, Germany

**Keywords:** Proteases, Renal pathophysiology, Complement system

## Abstract

Proteases play a central role in regulating renal pathophysiology and are increasingly evaluated as actionable drug targets. Here, we review the role of proteolytic systems in inflammatory kidney disease. Inflammatory kidney diseases are associated with broad dysregulations of extracellular and intracellular proteolysis. As an example of a proteolytic system, the complement system plays a significant role in glomerular inflammatory kidney disease and is currently under clinical investigation. Based on two glomerular kidney diseases, lupus nephritis, and membranous nephropathy, we portrait two proteolytic pathomechanisms and the role of the complement system. We discuss how profiling proteolytic activity in patient samples could be used to stratify patients for more targeted interventions in inflammatory kidney diseases. We also describe novel comprehensive, quantitative tools to investigate the entirety of proteolytic processes in a tissue sample. Emphasis is placed on mass spectrometric approaches that enable the comprehensive analysis of the complement system, as well as protease activities and regulation in general.

## Role of proteases in inflammation

Enzymatic proteolysis controls myriad physiological and pathophysiological processes, such as differentiation (Canalis et al. 2003), development (Kopan and Ilagan 2009), apoptosis (Taylor et al. 2008), hormone activation (Hampton 2002), neurodegeneration (O’Brien and Wong 2011), and cancer (Kessenbrock et al. 2010). Protease activity is essential for propagation and resolution of coagulation and inflammation. In inflammation, rapid protease activity is a key component of the innate immune system and contributor to the microenvironment and responsible for tissue remodeling. Several proteases are active within the inflammatory microenvironment, such as cathepsins (Joyce and Pollard 2009), urokinase PAR receptors (Andreasen et al. 1997; Joyce and Pollard 2009), matrix metalloproteinases (MMPs) (Prudova and Overall 2010; auf dem Keller et al. 2013; Eckhard et al. 2016), lysozyme (Satoskar et al. 2020), and the complement system (Ricklin et al. 2010). Recently, it has been shown that many of the proteases also target not only their direct substrates but also display unexpected substrates, modifying additional protein factors, which in turn interact with one another in a proteolysis-dependent manner. This hypothesis of a tightly regulated and fate-determining “protease web” (Fortelny et al. 2014; Rinschen et al. 2018b) postulates that proteases form functional networks with many interactions to govern pathophysiological processes. This notion expands the traditional and widely accepted concept of unidirectional proteolytic cascades, such as the initiation of apoptosis by caspase-8/-9-mediated proteolytic activation of caspase 3 (Porter and Jänicke 1999). With 588 and 628 proteases encoded in the human and mouse genome, respectively (Puente et al. 2003), the discovery of the complex interactions between these different proteolytic enzymes and systems offers a plethora of novel therapeutic cues for targeted intervention. These might remediate earlier failures that considered only a fraction of the activities of selected proteases in a very specific context.

The glomerulus is a key part of the kidney that maintains its filtration function. A significant fraction (> 115,000 prevalent patients in the US, 2017) of end-stage kidney disease is a result of glomerular diseases (USRDS 2019). Anatomically, the glomerulus consists of capillary loops and endothelia, mesangial cells, and podocytes (Dressler 2006). The close interaction with various immune cells (e.g., T_H_17 cells or CD3^+^ T cells) control glomerular function and phenotype (Krebs et al. 2017; Turner et al. 2018; Park et al. 2020). Molecular and signaling processes govern the progression of inflammatory glomerular disease. The presence of proteases is a hallmark of various forms of inflammatory, glomerular kidney diseases (Rinschen et al. 2018b), including roles for the inflammasome (Shahzad et al. 2015), the cathepsin family of proteases (Sever et al. 2007; Höhne et al. 2018; Merchant et al. 2020), lysozyme (Satoskar et al. 2020), MMPs (Zeisberg et al. 2006; Liu 2011), and the caspase system (Wang and Mitch 2014). In addition, the complement system, as part of the innate immune system, has emerged as an attractive target for glomerular diseases (Zipfel et al. 2019). This sets the stage for proteases as an increasingly appreciated but underexplored target for drug development in kidney disease, empowered by decades of successful protease inhibitor development targeting cardiovascular and infectious diseases (Drag and Salvesen 2010; Verhelst 2017).

The aims of this article are to (1) describe our latest understanding of pathomechanisms of proteolytic systems in inflammatory glomerular kidney disease, particularly focusing on the role of the complement system in lupus nephritis and membranous nephropathy, and (2) highlight novel proteomics strategies using state-of-the-art mass spectrometry for the study of protease function in the context of glomerular function in inflammatory kidney disease.

## The complement system, a key serum protease system

**Fig. 1:**
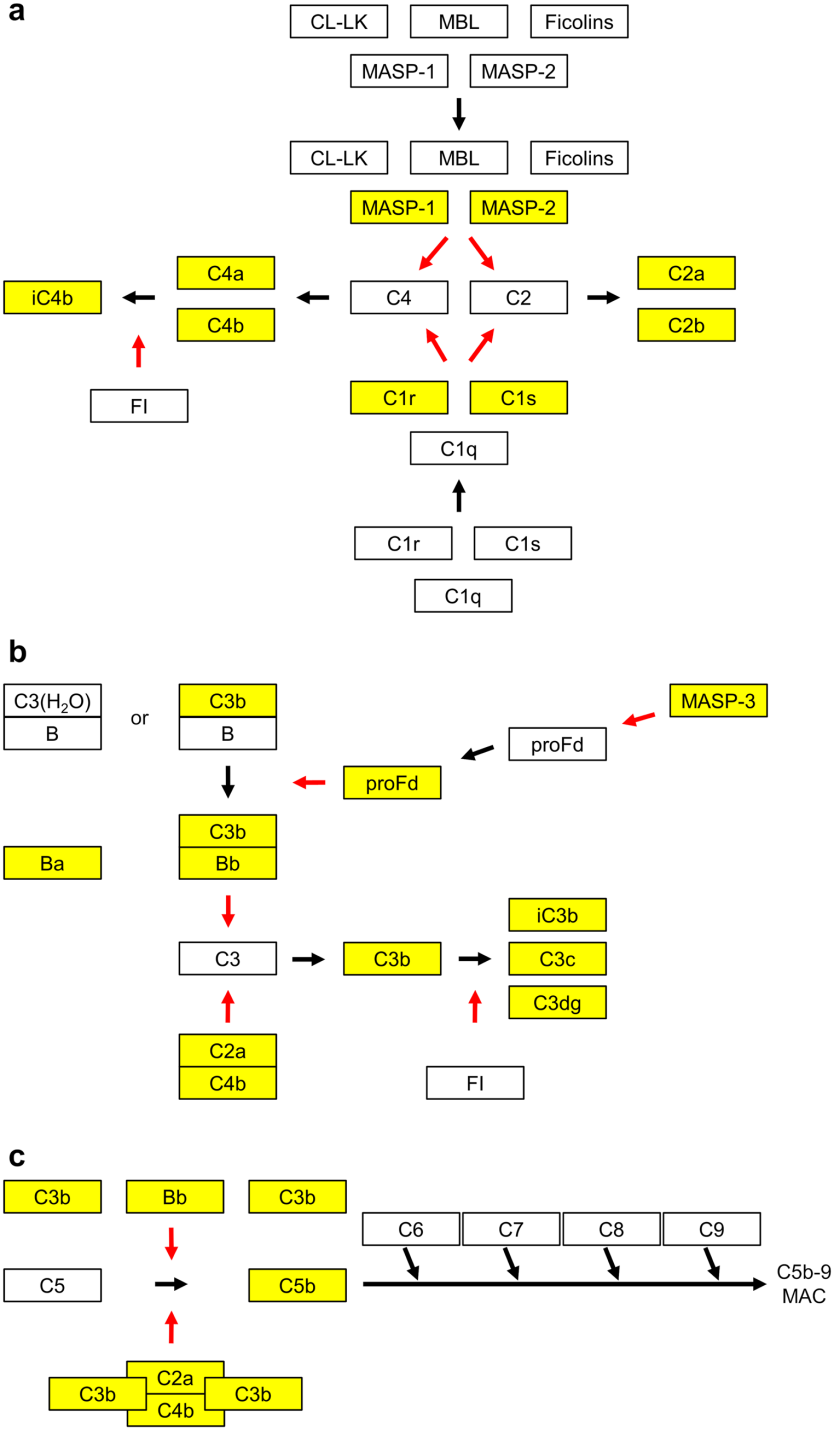
Overview of the complement system, a proteolytic system activated in inflammatory kidney disease. Cleaved complement proteins are colored yellow. Red arrows indicate enzymatic activity. **a** Two pathways that are initiated by pattern recognition molecules may lead to activation of the complement system. The lectin pathway is initiated when either one of the two collectins, mannose-binding lectin (MBL) or collectin-LK (CL-LK), or one of the three ficolins (H-ficolin, L-ficolin, and M-ficolin) recognizes microbial carbohydrates or modified self surfaces. The enzymes MBL-associated serine protease-1 and -2 (MASP-1 and MASP-2) that are attached to these recognition molecules get activated and now have the potential to cleave the C2 and C4 proteins into the fragments C4a and C4b and C2a and C2b, respectively. Similarly, the recognition molecule C1q that will initiate the so-called classical pathway may recognize deposited immunoglobulins bound to pathogens or apoptotic cells directly. When C1q binds, the two attached enzymes C1r and C1s may get activated and subsequently a cleavage of C2 and C4 may occur. **b** The central complement protein C3 may be activated through two enzymatic complexes: (1) spontaneous hydrolysis of the C3 thioester may form C3(H2O) that will bind factor B (B). This allows the enzyme factor D (D) to cleave B into the fragments Ba and Bb. This will allow Bb to cleave factor C3 into the fragment C3b. (2) similarly, if B binds to C3b it will be cleaved by D and lead to more generation of C3b—a positive feedback loop is formed. The fragments C4b and C2a generated via the lectin or classical pathway form complexes and this leads to C2a-mediated cleavage of C3 to C3b. The enzyme factor I (FI) will further process C3b to the fragments iC3b, C3c, and C3dg. The different fragments of C3 can bind to different receptors and initiate the activation of cells. **c** When more C3b molecules are deposited next to C4bC2a or next to C3bBb, a cleavage of C5 is initiated, leading to the fragments C5a and C5b. This will initiate the formation of a so-called membrane-attack complex consisting of C5b, C6, C7, C8, and C9 molecules. Such a complex may get inserted into membranes. The C5a molecule that was formed may bind to receptors and activate cells

The complement system is a well-studied and relevant proteolytic system whose activation is widely accepted to be triggered during glomerular kidney disease. The complement system is an essential part of the innate immune system and is vital for maintaining tissue homeostasis (Ricklin et al. 2010; Bajic et al. 2015). It can identify and opsonize targets, including invading microbes, immune complexes, necrotic tissue, and apoptotic cells, and hereafter facilitate their safe removal via phagocytosis (Merle et al. 2015b). The proteolytic cascades of the complement system are tightly regulated (Fig. 1) by several proteins (Merle et al. 2015a; Schmidt et al. 2016). If the delicate balance between activation and regulation is tipped, the system may act as a double-edged sword causing self-damage manifesting as various immune-mediated and inflammatory diseases (Bajic et al. 2015).

Initiation of the complement system may occur through three pathways (Fig. 1), termed the classical pathway (CP), lectin pathway (LP), and alternative pathway (AP). While the CP and LP have specific initiating molecules (antibodies bound to antigens and patterns of carbohydrate structures, respectively), the AP is triggered by the spontaneous activation of complement factor C3 in the fluid phase. The pathways converge at the cleavage of complement factor C3 into C3b and C3a, resulting in (1) opsonization of pathogens by split products of C3, (2) cell lysis via formation of the membrane attack complex, and (3) inflammation by recruitment of inflammatory cells such as neutrophils by pro-inflammatory mediators like C5a (Merle et al. 2015a).

While it is widely acknowledged that the complement system plays an integral role in disease progression and can guide clinical diagnosis and classification, there is a lack of understanding of which complement proteins or functional protein fragments, also termed proteoforms (van der Burgt and Cobbaert 2018), are best suited as sensitive and specific diagnostic biomarkers when measured as part of routine clinical care in inflammatory kidney disease. The complexity of the complement system, which encompasses approximately 50 protein in circulation, demands a holistic and quantitative approach to identify the most important contributors and markers of inflammation (Ricklin et al. 2010). For an overview of appropriate measurement strategies, we refer to other reviews (Ekdahl et al. 2018). The most commonly used assays are nephelometry and turbidimetry which utilize polyclonal antibodies against a specific analyte (e.g., C3 or C4). Notably, comprehensive approaches regarding the high-throughput profiling of clinical samples are currently missing.

Clinical trials with complement inhibitors such as CCX168 targeting C5aR (clinical trial code NCT02994927), OMS721 targeting MASP2 (NCT03608033), or C1INH targeting C1r and C1s (NCT02547220) are currently under investigation and may potentially be integrated as new treatments of selected diseases. Currently, 28 clinical trials, including six phase III trials, all with relevance to glomerular kidney disease, are ongoing and the C5 inhibitor Eculizumab is already available on the market (Zipfel et al. 2019).

## The complement system in lupus nephritis

**Fig. 2:**
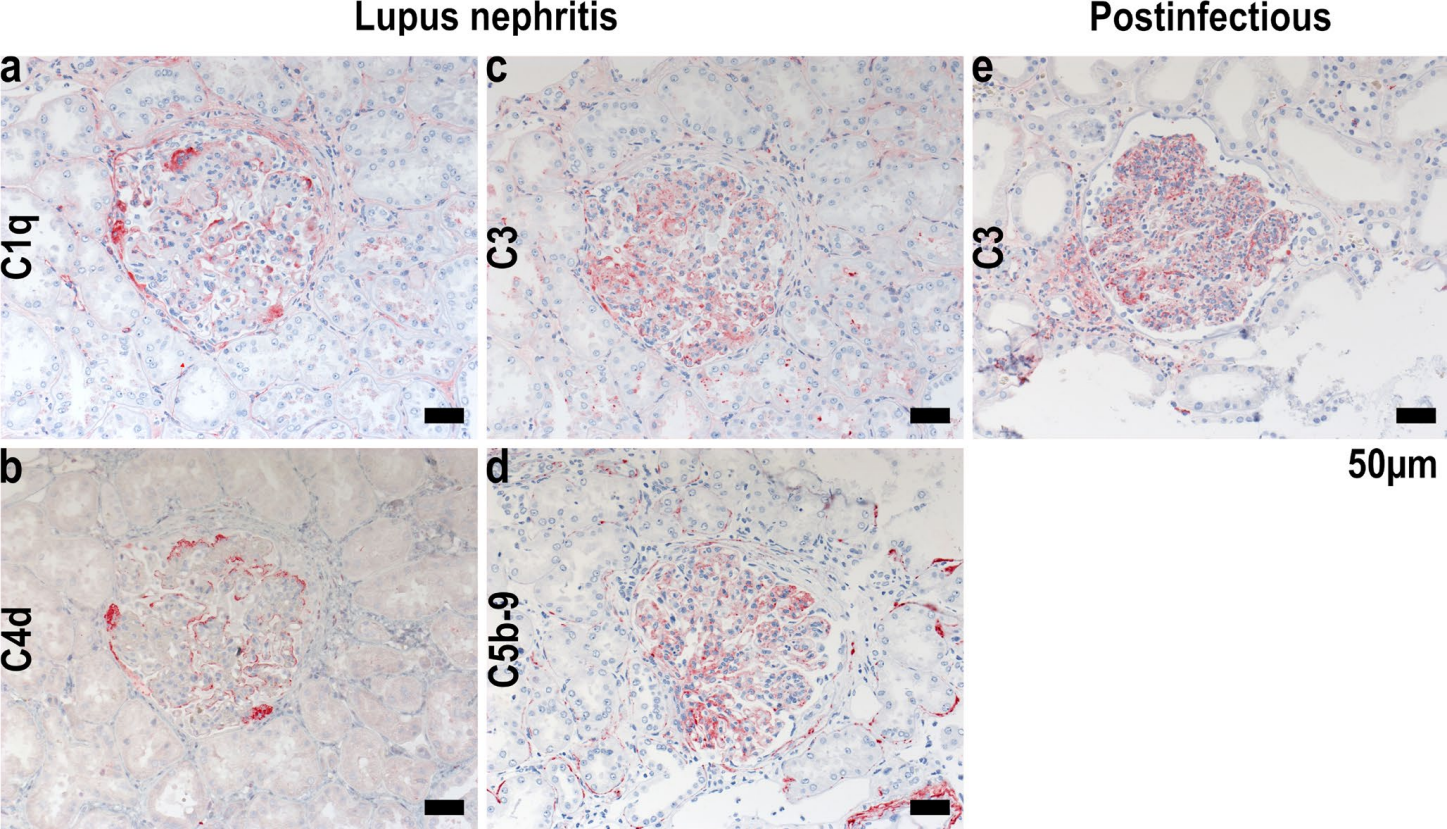
Complement staining in inflammatory glomerular disease. Immunostaining of complement components in human lupus nephritis (**a-d)** and postinfectious glomerular nephropathy (**e**)

Complement proteins are found in many patients presenting lupus nephritis (LN) and this upregulation is a hallmark of the disease. The so-called “full-house” immunofluorescent staining pattern carried out as the gold-standard clinical testing in lupus nephritis biopsies with colocalization of IgG, IgM, IgA, C1q, and C3 (C4) is almost solely seen in lupus nephritis (Gianviti et al. 1999) in contrast to other glomerular kidney diseases (Fig. 2a-d, e). The traditional view of complement activation in the glomerulus of patients with LN is through activation of the classical pathway initiated via binding of C1q to immune-complex depositions in the glomeruli (Berden et al. 1999; Person et al. 2020). When deposited in the mesangium and subendothelial space, the immune complexes are proximal to the glomerular basement membrane and in direct contact with the systemic circulation (Bomback et al. 2016).

Activation of the classical complement pathway generates C3a and C5a, which causes influx of neutrophils and mononuclear cells, which is the pattern seen in the proliferative LN subtypes (LN III and IV) (Weening et al. 2004). The injury in class V LN (or membranous LN) is limited to the glomerular epithelial cells. Complement activation in membranous LN appears to be dominated by activation of the Lectin pathway (Ma et al. 2013). The alternative pathway amplifies the activation initiated through either of the classical or the lectin pathway (Fig. 1a). Key component of the AP is mannan-binding lectin-associated serine protease 3 (MASP-3), which is the only known activator of factor D and therefore essential in the AP pathway (Pihl et al. 2017), that was found at lower concentration in the plasma of patients with LN compared with lupus patients without nephritis (Troldborg et al. 2018). In LN kidney biopsies, properdin depositions in glomeruli were associated with increased proteinuria, and factor B depositions were associated with longer disease duration and interstitial fibrosis development (Sato et al. 2011). Blockade of the alternative and the lectin pathway through inhibition of MASP-1 and MASP-3 ameliorated LN in a mouse model (Machida et al. 2018).

## The complement system in membranous nephropathy

Membranous nephropathy (MN) is an antibody-mediated proteinuric kidney disease. Glomerular complement deposition can be readily detected by immunofluorescence- and mass spectrometry-based approaches in patient biopsies (Person et al. 2019; Ravindran et al. 2020). The proposed pathophysiological mechanism of MN derives from investigations in a rat model of the disease, the so-called passive Heymann nephritis (PHN) (Heymann 1952). In this model, podocyte-directed heterologous antibodies from sheep (or other species) are transferred to rats, causing the formation of subepithelial immune deposits, which are considered the morphological hallmark sign of MN and proteinuria. The injected antibodies induce the local activation of the complement system with formation of the membrane attack complex C5b-9 (Kerjaschki 1992). In PHN, blocking the complement system by means of cobra venom factor was reported to completely prevent proteinuria development (Salant et al. 1980). However, other experimental reports described the development of MN in the absence of complement deposition (Tomas et al. 2016, 2017) and after pharmacological complement depletion (Leenaerts et al. 1995), challenging the concept of the complement system as the sole mediator of cell injury and proteinuria in MN.

In patients with MN, autoantibodies against two podocyte antigens have been identified, the phospholipase A2 receptor 1 (PLA2R1) and thrombospondin type-1 domain-containing 7A (THSD7A) (Beck et al. 2009; Tomas et al. 2014). The classical pathway of the complement system is activated by binding of an antibody to an antigen. This mechanism can in principle apply for an antibody-mediated disease such as MN. However, anti-PLA2R1 and anti-THSD7A autoantibodies are dominantly of the IgG4 subclass, which is the IgG subclass with the least C1q binding capacity (Vidarsson et al. 2014), indicating that the alternative and lectin pathways may play a role in the pathogenesis of MN (Seikrit et al. 2018; Zhang et al. 2020). However, patients with PLA2R1- and THSD7A-associated MN usually have autoantibodies of C1q-binding non-IgG4 subclasses as well, principally enabling the activation of the complement system via the classical pathway (Huang et al. 2013; von Haxthausen et al. 2018). A study published while this paper was in review showed that IgG4 glycosylation in PLA2R1-associated MN may be responsible for activation of the lectin pathway, and a subsequent activation of podocyte proteolytic pathways via cathepsin proteases (Haddad et al. 2020).

Taken together, the presence of complement components at the site of tissue injury is undoubted in MN, but whether this contributes to MN pathogenesis or simply represents an epiphenomenon is still unclear today. Novel methodological approaches are needed to clarify the role of complement in MN.

## Novel analytical approaches to map proteolysis in vivo

In order to prioritize and stratify patients for guiding treatment with complement inhibitors during the course of an immunosuppressive therapy, it would be beneficial to determine the activation state of proteolytic systems in patients with LN or MN to improve clinical care. To this end, several mass spectrometry-based approaches have been developed that are currently being transferred to preclinical disease models, awaiting further validation for application in clinical practice (Huesgen et al. 2014). In the following paragraph, we will review novel strategies for profiling and mapping proteolytic systems, including the complement system, using innovative analytical mass spectrometric technologies.

Proteomic profiling by nano-flow liquid chromatography tandem mass spectrometry (nLC-MS/MS) can be used to detect and quantify the entirety of proteases within the glomerulus to gain insight of the intricate protease-networks, making this an indispensable tool for understanding kidney disease and defining novel markers of kidney inflammation. Modern proteomics has matured to provide deep maps of protein compositions (Rinschen et al. 2018a) and is able to be paired with high-throughput automation (Müller et al. 2020) and quality control systems important for clinical application (Dayon et al. 2014). Alternatively, routine clinical assaying to quantify specific proteases as protein markers, upregulated in response to disease, can be performed with immunometric assays (Kapprell et al. 2011). While the qualification and quantification of proteases are indispensable, full characterization of protease networks also requires functional assessment of protease activity. In order to correlate protease concentration to protease activity, the relationship between concentration and function can be established using activity-based protein profiling (ABPP), a method to identify the enzymatically active proteases in a sample (Cravatt et al. 2008). For ABPP, chemical probes are designed to target specific proteases, mainly cysteine and serine proteases but rarely metalloproteases due to their lack of stable acyl-enzyme intermediates (van Kasteren et al. 2017). ABPP probes generally consist of three elements: (1) a reactive group which will specifically bind to the proteases of interest, mostly at the active site; (2) a reporter tag for purification (e.g., biotin); and (3) a linker to avoid steric hindrance by the reporter tag. Upon binding of the probes to the proteases in the sample, the reporter tag can be utilized to enrich the labeled proteases by affinity purification (e.g., by streptavidin beads for biotin-labeled probes). Subsequently, the purified proteases are trypsinized for identification by nLC-MS/MS (Chen et al. 2017). Additionally, ABPP can be utilized in combination with multiplexing assays on CyTOF instrumentation (Poreba et al. 2020; Savickas and auf dem Keller 2017). For detailed investigations on protease maturation itself, e.g., zymogen removal, a targeted degradomics approach can be used (Savickas and auf dem Keller 2017), especially for complement activation.

In addition to monitoring the proteases themselves, several elegant approaches for the identification and quantification of the proteolytically processed proteins on a global scale have been developed, providing insight into novel protease targets and networks (Rinschen et al. 2018c; Rinschen and Saez-Rodriguez 2020). These analytical strategies build on enrichment of endogenous protein N-termini, partially those N-termini that result from endogenous proteolytic activity (e.g., through complement system activity). This enrichment step prior to analysis by nLC-MS/MS is required because the excess of peptides generated during standard trypsinization in conventional proteomics workflows would be indistinguishable from peptides generated by in vivo proteolysis. These novel approaches thus alleviate some of the limitations of classical proteomics workflows to aid the discovery and quantification of endogenously formed N-terminal peptides (Niedermaier and Huesgen 2019).

**Fig. 3:**
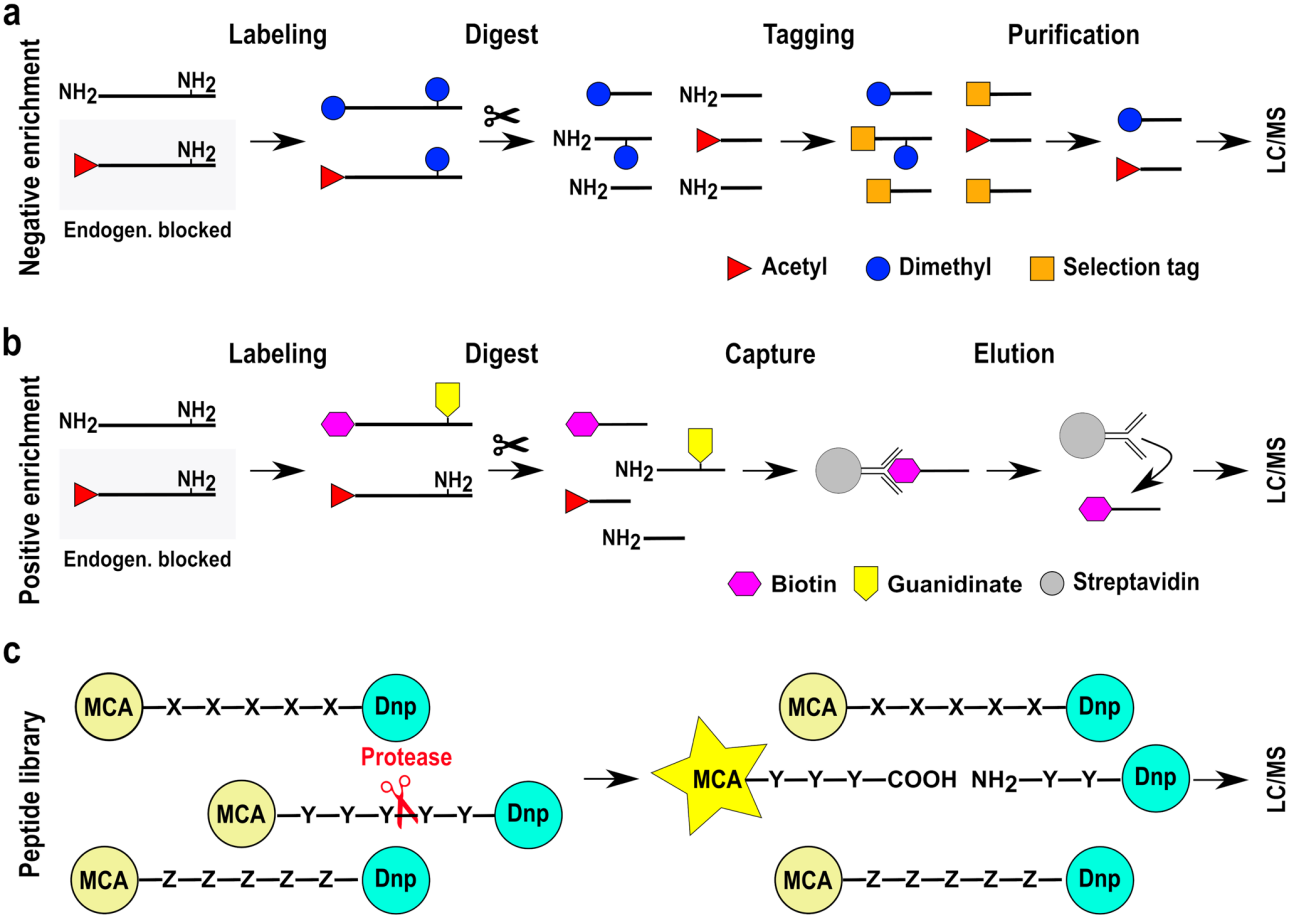
Selected mass spectrometry-based methods for the investigation of N-termini. **a** Negative enrichment approaches (e.g., TAILS, HUNTER, and CoFRADIC) chemically label free N-termini but not endogenously modified N-termini. All N-termini subsequently generated through trypsin digestion feature a free N-terminus, whereas all natural N-termini are protected due to chemical labeling (e.g., dimethylation) or endogenous modifications (e.g., acetylation). The trypsin-generated, free peptide N-termini are tagged with a selection tag (e.g., HPG-ALD, undecanal) and can be separated from the sample, effectively enriching for endogenously modified or protected N-termini. Please note that the “selection tags” are different chemical moieties dependent on the specific protocol used. **b** Positive enrichment methods purify N-termini by affinity enrichment. Protein N-termini are labeled by guanidination/biotinylation in a two-step labeling reaction. Biotin-labeled N-termini are purified with streptavidin beads, and the bound N-terminal peptides are eluted off the beads. **c** An alternative strategy relies on use of synthetic peptide libraries as targets for endogenous proteases. The pentapeptides are coupled to a fluorescence resonance energy transfer (FRET) pair consisting of 7-methoxycoumarin-4-acetamide (MCA) as a fluorophore and dinitrophenyl (DNP) as a quencher. Upon protease cleavage at any of the amide bonds, the quenching is alleviated, and MCA emits fluorescence at 405 nm providing information on protease activity in a given sample. After detection of protease activity, subsequent steps are required to identify the proteases by LC/MS

Several approaches for the enrichment of endogenous N-termini have been developed: negative selection enrichment, such as terminal amine isotopic labeling of substrates (TAILS) (Kleifeld et al. 2010; Savickas et al. 2020), combined fractional diagonal chromatography (COFRADIC) (Gevaert et al. 2003), or High-efficiency Undecanal-based N Termini EnRichment (HUNTER) (Weng et al. 2019) utilize chemical labeling to facilitate enrichment (Fig. 3a). Chemical labeling of endogenous N-termini prior to trypsinization, by reductive dimethylation (Boersema et al. 2009; Demir et al. 2017), acetylation (Gevaert et al. 2003), or alternatively TMT (Savickas and auf dem Keller 2017; Savickas et al. 2020), distinguishes native protein N-termini from N-termini generated through the trypsin digestion. The endogenous, free N-termini are chemically labeled prior to digestion with trypsin, and thus, all endogenous, original N-termini are either naturally modified (e.g., N-terminal acetylation) or chemically labeled (dimethylation or TMT). Subsequently, the proteolytic digest of proteins with trypsin generates small peptides, which all feature a free N-terminus and are present in high excess of the natural N-termini. These residues are then chemically tagged with a second compound that is different from the chemical labeling compound employed in the first step. This second tagging enabled depletion of the confounding and highly abundant tryptic peptides and yields the complete set of all N-termini in the sample, termed N-degradome.

Different labeling and tagging compounds are available for the enrichment of N-termini, each with their inherent properties (Fig. 3a): TAILS utilizes hyperbranched polyglycerol-aldehydes of high molecular weight (HPG-ALD) for separation by filtration through a 30 kD MWCO units, COFRADIC uses 2,4,6-trinitrobenzenesulfonic acid (TNBS) for separation by chromatographic properties, and HUNTER applies the aldehyde undecanal for easy depletion by hydrophobic retention on C_18_ material. All these tags used for the second tagging step are highly reactive towards the free N-terminus generated by tryptic digest. The chemically tagged peptides are depleted, and the remaining, endogenous N-termini used for subsequent LC/MS analysis. Quantitative alterations in the proteolytic network can thus be resolved via dimethyl (Demir et al. 2017) or TMT labeling strategies (Kleifeld et al. 2011). Modified protocols of TAILS (Schilling et al. 2010) or COFRADIC (Canbay and auf dem Keller 2021) can be used for profiling of C-termini which is especially important for corroborating findings of N-termini proposed to result from endo-peptidase activity.

In contrast to these workflows, positive enrichment strategies enrich endogenously modified or chemically protected protein N-termini by affinity enrichment to retain desired N-termini containing peptides (Fig. 3b). A multi-step labeling procedure targets at first lysine residues for guanidination at high pH and, secondly, protein N-termini for biotinylation at neutral pH. Subsequently, the trypsin digest is carried out yielding peptides with free, non-biotinylated N-termini. Separation of biotinylated endogenous N-termini that are from non-biotinylated tryptic peptides is carried out using streptavidin beads (Timmer et al. 2007). A slightly different positive selection approach employs the enzyme subtiligase to ligate short peptides with a tag for enrichment, most frequently a click-chemistry functional moiety, to free N-termini present in the sample (Weeks and Wells 2020). In both cases, the eluted N-termini are subsequently identified by nLC-MS/MS.

An elegant method to measure proteolytic activity in a given sample and gaining substrate specificity information utilizes artificial peptide libraries (Fig. 3c) (Sun et al. 2007; Fields 2010). A FRET system consisting of 7-methoxycoumarin-4-acetamide (MCA) and dinitrophenyl (DNP) is conjugated to an artificial pentapeptide library containing up to 2.47 million different protease substrate sequences. The fluorescent labels only fluoresce if the pentapeptide is proteolyzed (Enari et al. 1996). Following cleavage of the pentapeptide by a protease, the quencher DNP is removed, and MCA emits fluorescence, which can be quantified (Enari et al. 1996; Kapprell et al. 2011). Addition of different protease inhibitors enables to pinpoint the protease activity to specific protease classes. As this method relies solely on the detection of a fluorescence signal rather than LC/MS, subsequent proteomics experiments are required to identify the proteases responsible for the cleavage. A very similar approach is the hybrid combinatorial substrate library (HyCoSuL) (Poreba et al. 2017). This method utilizes a combination of unnatural and natural amino acids in artificial, fluorogenic peptide substrates. A HyCoSul library has been successfully applied in determination of the extended substrate preferences for the SARS-CoV-2 main protease (Rut et al. 2020).

## Global proteolytic analysis in glomerular disease

In the following paragraph, we will review the application of novel analytical techniques and the key insights gained from them.

One hallmark of glomerular inflammatory kidney disease is nephritis and proteinuria. When the renal filtration barrier becomes leaky, proteins leak into the urine (proteinuria). These proteins may also contain active proteases. From studies chiefly in nephrotic syndrome, it has been suggested that these proteases contribute to uncontrolled cleavages of ion transporters and channels, with functional consequences (Artunc et al. 2019). For instance, one target is the epithelial sodium channel (ENaC, *SCNN1*), a heterotrimeric sodium channel which is often targeted by aldosterone antagonists and channel inhibitors (e.g., amiloride). It has been suggested that ENaC cleavage leads to increased sodium retention and that the proteolytic cleavages are mainly carried out by serine proteases (Svenningsen et al. 2009). Serine proteases have been reported at elevated concentration in urine due to aberrant filtration and could detrimentally affect processes such as ENaC-dependent sodium reabsorption in the distal tubule. Additional proteases found to be active and at elevated concentration in the urine are plasmin (Svenningsen et al. 2009) and prostasin/kallikrein (Zachar et al. 2015). The Artunc group has catalogued the proteases active in urine from patients with acute nephrotic syndrome and a corresponding mouse model (Wörn et al. 2021) by nLC-MS/MS and in a fluorescence-based assay (Kapprell et al. 2011) (Fig. 3c). Mainly serine proteases of the coagulation and complement cascade could be identified in the nephrotic syndrome urine. Their activity could be inhibited by using the serine protease-specific inhibitors AEBSF and aprotinin. By capturing those active serine-proteases with the help of AEBSF-coupled beads, the main active serine proteases could be identified as plasminogen, factor VII-activating protease, coagulation factor XIII, and the complement factors D and B. The identification of plasminogen as the main serine protease in the nephrotic syndrome urine is in line with similar reports of plasminogen/plasmin as biomarkers for glomerular injury (Egerman et al. 2020). In contrast, the urine protease composition in healthy controls was limited to low molecular weight and locally expressed proteases like kallikrein-1 or neprilysin (Wörn et al. 2021).

A preclinical model was used to analyze proteolytic processing in a model of cisplatin-induced acute kidney injury (Späth et al. 2018). The authors recorded proteome, transcriptome, and N-degradome from the same animals. N-degradomic analysis revealed coverage of 1865 N-termini from 1166 unique proteins. These included the N-termini of the entire complement system, covering the classical, lectin, and alternative pathways. Major sites of action included the complement components C3 and C4, each with four unique N-terminal processing sites and an arginine-specific cleavage motif in C4. Corresponding upstream components were also regulated, e.g., cathepsin L1/2 and complement factor D. These data indicate that comprehensive complement mapping in the tissue is possible and that this may be useful to accurately quantify the activation of protease systems using minimal amount of patient sample if combined with single-nephron proteomics techniques (Höhne et al. 2018). Comprehensive glomerular kidney disease degradomics datasets have been generated as well, suggesting proteolytic processing of ACTN4, podocin, and several other proteins responsible for podocyte maintenance, as well as complement proteolysis (Rinschen et al. 2017).

### Conclusion

Proteases are key modulators of glomerular function, and the complement is an important proteolytic system that communicates between the epithelia and the innate immune system. While several inflammatory kidney diseases show that proteolysis is active and can be targeted genetically, it remains under investigation—both clinically and preclinically—if protease inhibition can emerge as therapeutic strategy in glomerular inflammatory kidney disease.

Within this context, novel aspects of complement system characterization can be useful. Traditionally regarded as a simple proteolytic cascade, the complement system exhibits increasingly recognized complex interactions with other proteolytic enzymes and inhibitors (auf dem Keller et al. 2013), resulting in severe challenges for the development of reliable parameters for complement-based diagnosis and patient stratification in kidney disease. Part of the current limitations are analytical in nature, given the fact that more than 50 proteins, each with multiple proteoforms with distinct function and widely different abundance, make up the complement system. No consensus has been reached on what to measure, when to measure and how to measure complement activation (Ekdahl et al. 2018), and non-canonical effects of complement proteases have not yet been systematically analyzed in kidney disease. Further proteolytic systems, such as coagulation and fibrinolysis, on the other hand, are not commonly investigated despite possible interactions (Amara et al. 2010; Oikonomopoulou et al. 2012). Therefore, further improvement of mass spectrometry-based and chemical biology techniques is needed to further and deeper profile proteases’ action in inflammatory kidney disease. Analysis of the proteolytic microenvironment in glomerular disease, including the complement system, may help stratify patients for therapeutic intervention, for instance, by complement inhibitors. Candidates for deep proteolytic analysis by proteomics include membranous nephropathy and lupus nephritis. Here, integrated proteomics profiling of human kidney biopsies and serum samples will lead to an increased understanding of pathobiology of protease-driven inflammation and might be used to stratify and prioritize patients for therapy with complement inhibition.
